# Catalytic Degradation of Ciprofloxacin in Aqueous Solution by Peroxymonosulfate Activated with a Magnetic CuFe_2_O_4_@Biochar Composite

**DOI:** 10.3390/ijms24065702

**Published:** 2023-03-16

**Authors:** Youmei Zeng, Guangming Zhou, Dandan He, Guilong Peng

**Affiliations:** 1 School of Chemistry and Chemical Engineering, Southwest University, Chongqing 400715, China; 2 State Key Laboratory of Silkworm Genome Biology, Key Laboratory of Sericultural Biology and Genetic Breeding, Ministry of Agriculture and Rural Affairs, College of Sericulture, Textile and Biomass Sciences, Southwest University, Chongqing 400715, China

**Keywords:** magnetic copper ferrite, biochar, catalytic performance, ciprofloxacin, peroxymonosulfate

## Abstract

A magnetic copper ferrite and biochar composite (CuFe_2_O_4_@BC) catalyst was prepared by an improved sol-gel calcination method and initially used for the removal of antibiotics ciprofloxacin (CIP) by activated peroxymonosulfate (PMS). Using CuFe_2_O_4_@BC as the activator, 97.8% CIP removal efficiency could be achieved in 30 min. After a continuous degradation cycle, CuFe_2_O_4_@BC catalyst still exhibited great stability and repeatability and could also be quickly recovered by an external magnetic field. Meanwhile, the CuFe_2_O_4_@BC/PMS system presented good stability for metal ion leaching, which was far less than the leaching of metal ions in the CuFe_2_O_4_/PMS system. Moreover, the effects of various influencing factors, such as initial solution pH, activator loading, PMS dosage, reaction temperature, humic acid (HA), and the inorganic anions were explored. The quenching experiments and the electron paramagnetic resonance (EPR) analysis manifested that hydroxyl radical (•OH), sulfate radical (SO_4_^•−^), superoxide radical (O_2_^•−^), and singlet oxygen (^1^O_2_) were generated in the CuFe_2_O_4_@BC/PMS system, while ^1^O_2_ and O_2_^•−^ are mainly involved in the degradation process. The synergistic effect between CuFe_2_O_4_ and BC enhanced the structural stability and electrical conductivity of the material, which promoted the bonding between the catalyst and PMS, resulting in the enhanced catalytic activity of CuFe_2_O_4_@BC. This indicates that CuFe_2_O_4_@BC activating PMS is a promising remediation technique for CIP-contaminated water.

## 1. Introduction

Recently, water contamination caused by the excessive use and improper treatment of antibiotics has obtained increasing attention [1,2], and owing to their poor biodegradability and high toxicity, the majority of antibiotics are hardly eliminated by traditional methods such as biotechnology [3,4]. As a typical and widely used antibiotic, ciprofloxacin (CIP) increases antibiotic resistance of bacteria in wastewater and poses a serious threat to humans and aquatic organisms [5]. Therefore, it is imperative to develop an efficient technology to remove residual antibiotics from contaminated water environments.

A variety of methods have been explored to treat antibiotic pollutants such as biotransformation, adsorption, electrocoagulation method [6,7], and advanced oxidation processes (AOPs). Additionally, different types of highly effective AOPs have been widely studied due to their high oxidation ability to various pollutants, such as Fenton processes [8], UV-photolysis driven processes [9], ozonation [10], and sulfate radical-based AOPs (SR-AOPs) [11,12,13]. Notably, the SR-AOPs have received increasing concerns due to the generation of sulfate radical (SO_4_^•−^) with high reactivity [14,15]. Nonetheless, when in direct contact with contaminants, the unactivated peroxymonosulfate (PMS) results in a poor degradation efficiency, which restricts its practical application. 

Recently, mixed metal catalysts have attracted great interest in the activation of PMS due to their versatility, stability, and better catalytic activity. Such as Fe(II), Cu(II), Co(II), Ag(I), Ni(II), and Mn(II), which have been explored to activate PMS for pollutant degradation [16], and the Co(II) shows strong activity in initiating PMS to generate sulfate radicals to degrade pollutants [17]. However, due to the toxicity of Co(II), it is not conducive to the practical application of wastewater treatment. Additionally, Cu(II) is not currently considered as a potential carcinogen, and copper-based oxides are highly stable, efficient, and recyclable [18]. Presently, the synthesis of spinel ferrite with Fe^3+^, a potential PMS catalyst, which could synthesize a relatively stable structure and keep a lower metal ion leaching. Another advantage of the spinel ferrite is its magnetism, which makes it easy to separate from water [16]. As a typical representative of spinel ferrite, CuFe_2_O_4_ has attracted great interest in the heterogeneous activation process of organic pollutant degradation [19,20]. However, due to its strong magnetism, copper ferrite particles will aggregate, resulting in a decrease in its electron transfer ability, thereby, reducing its PMS activation activity [16]. Thus, a variety of support materials for the preparation of spinel ferrite composites (SFCs) have been studied to overcome these drawbacks, such as biochar, graphene oxide, metal-organic frameworks, and a variety of porous transition metal oxides. For example, Hao et al. proved that CuFe_2_O_4_-rGO had a better degradation efficiency than CuFe_2_O_4_ [21]. Nevertheless, the application of nanomaterials is severely restricted by the high preparation cost [22]. Thus, developing an environmentally friendly, low-cost, and widely applicable technology to improve catalytic efficiency is necessary.

Every year, a large number of agricultural wastes related to agricultural production are discarded, of which crop straw accounts for more than 50%, and its safe treatment and utilization have become a huge challenge. Traditional treatment methods (such as incineration) cannot effectively recover resources, but also lead to serious air pollution. In recent years, the development of green cleaning and resource utilization technology can convert agricultural waste into value-added organic fertilizer and biological energy, which brings great prospects for the “win–win” strategy. Among them, the conversion of carbon-rich biomass to value-added biochar (BC) is an attractive option. Due to its low cost and environmental friendliness, it has been extensively studied as a supporting matrix for various catalysts and proved to synergistically improve the catalytic performance [23,24]. Furthermore, it has rich defect structures and large specific surface area, remarkable electronic conduction ability, and abundant oxygen-containing functional groups [23]. BC shows excellent potential to increase the particle dispersion of spinel ferrite catalysts and improve electron transport in heterogeneous activation systems [25], which makes BC suitable to be the supporting material for catalyst loading. Zhao et al. reported that CuFe_2_O_4_@BC composites can effectively activate persulphate (PS) to degrade nitrochlorobenzene in soil [26]. Therefore, BC might be developed as a cost-effective, easily prepared, and environment-friendly supporting material. 

Mulberry is an important economical crop with versatile applications. However, the leaves of mulberry after harvesting and mulberry branches are mostly used as firewood or discarded as agricultural waste. It is worth noting that if a large number of mulberry branches can be converted into prospective biomaterials through appropriate biomass energy routes, it will have important theoretical guiding significance for environmental protection and the comprehensive utilization of agricultural mulberry branch resources [27]. Inspired by the above studies, mulberry branches biochar as a supporting material for ferrite matrix may also be an efficient catalyst for PMS activation in wastewater. The potential of mulberry branches biochar and its spinel ferrite composites for the removal of organic contaminants has not been explored so far and the relevant mechanism of material catalytic efficiency enhancement also requires to be comprehensively explained. 

Hence, the current study of mulberry branches biochar was prepared, then the prepared mulberry branches biochar was used as a base material to support magnetic copper ferrite (CuFe_2_O_4_) by an improved sol-gel combustion reaction to obtain an advanced composite catalyst (CuFe_2_O_4_@BC). The prepared material was applied to activate PMS for organic pollutants degradation and the CIP was selected as the target contaminant. Moreover, various characterization methods were used to examine the morphology, crystal structure, and structural characteristics of the produced catalysts. The effects of initial pH, catalyst dosage, PMS concentration, temperature, inorganic anions, and humic acid (HA) in the CuFe_2_O_4_@BC/PMS system were also investigated. Additionally, the potential applications of catalysts were evaluated in terms of magnetism, reusability, and stability. Finally, the degradation mechanism of the free radical and non-free radical processes was investigated by the XPS analysis, free radical quenching experiment, and EPR detection. Accordingly, this research is dedicated to exploiting biochar and developing a novel magnetic catalyst to achieve the effective removal of pollutants. 

## 2. Results and Discussion

### 2.1. Catalysts Characterization

**Figure 1:**
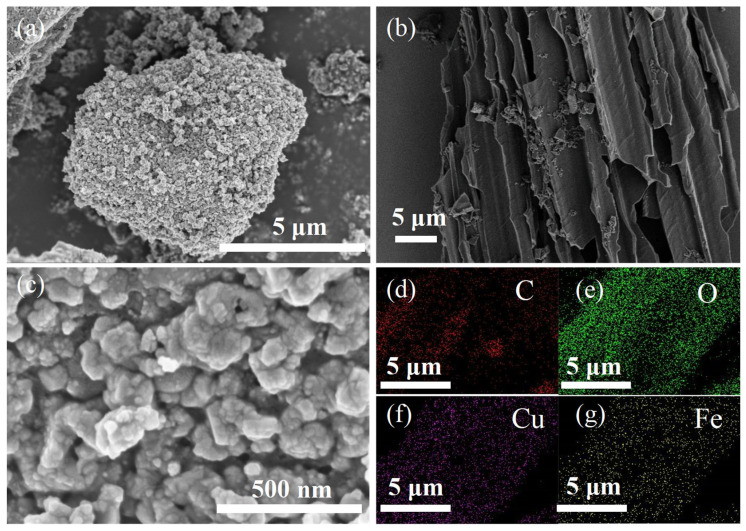
(**a**,**c**) SEM images and (**d**–**g**) EDS mapping images of the CuFe_2_O_4_@BC catalyst, (**b**) SEM images of BC catalyst.

To determine whether the loading of CuFe_2_O_4_ on the BC was successful, SEM images were characterized with prepared samples (Figure 1a–c). According to the comparison of the CuFe_2_O_4_@BC catalyst (Figure 1a) and the BC catalyst (Figure 1b), it could be seen that mass particles adhered to the surface of the BC, which were aggregated by the magnetic attraction of the ferrite according to previous reports [28,29].Meanwhile, the diameters of copper ferrite particles ranged from 50 to 200 nm, according to the TEM images of copper ferrite particles presented in Figure S1. EDS was employed to clarify the composition of the CuFe_2_O_4_@BC catalyst (Figure S2) and the mapping profiles of C, O, Cu, and Fe elements (Figure 1d–g), confirming that four elements were evenly distributed in the material. 

**Figure 2:**
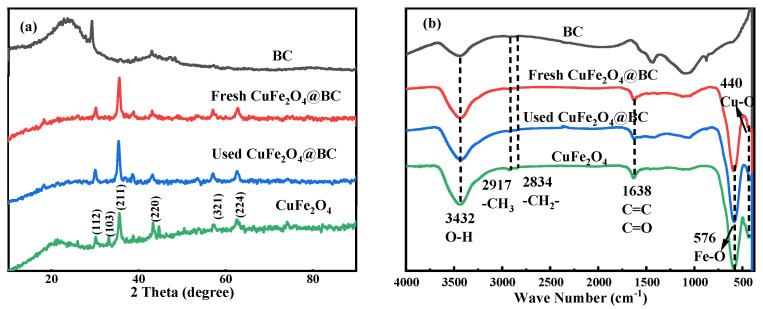
(**a**) XRD patterns and (**b**) FT–IR spectra of the prepared catalysts.

The crystal structures of BC, CuFe_2_O_4_, as well as the prepared CuFe_2_O_4_@BC catalysts, were obtained by XRD analyses, as displayed in Figure 2a. The great crystallinity of the catalysts could be seen in the diffraction diagram. For CuFe_2_O_4_, the characteristic peaks of XRD patterns at 29.91°, 34.72°, 35.16°, 43.77°, 57.03°, and 62.16° correspond to (112), (103), (211), (220), (303), and (224) planes of CuFe_2_O_4_ (PDF#34-0425), respectively, which revealed the synthesis of spinel CuFe_2_O_4_. Simultaneously, the XRD characteristic peak of BC was 26°~28°, and after loading, the main wide diffraction peak of BC (2θ = 26°) [30] was also identified in CuFe_2_O_4_@BC materials, representing that the structure of BC was not ruined during the formation process. Nevertheless, this peak came to be less evident, which might be owing to the diffraction intensity of BC being much weaker than the resulting CuFe_2_O_4_@BC catalysts [31]. Additionally, compared with the fresh CuFe_2_O_4_@BC composite, no extra peak was detected in the XRD spectra of the used CuFe_2_O_4_@BC composite, revealing that the composition and structure of the catalyst remained essentially unchanged after degradation.

The FT–IR spectrum analysis of pure BC, CuFe_2_O_4_, as the prepared and used CuFe_2_O_4_@BC catalyst, were shown in Figure 2b. For pristine CuFe_2_O_4_@BC, the absorption bands at 480 cm^−1^ and 570 cm^−1^ were due to the stretching vibration of the Cu–O bond [32] and verified the formation of the Fe–O bond with tetrahedral geometry [33], which illustrated the formation of Fe(III) and Cu(II) in tetrahedral and octahedral coordination environments, respectively. On the other hand, the C=C, C=O, and O-H stretching vibration peaks corresponding to the absorption peaks at 1500 cm^−1^, 1700 cm^−1^, and 3470 cm^−1^ could be observed in pure BC; the existence of rich oxygen functional groups on BC was confirmed, which was essential for the catalyst to maintain hydrophilicity and high dispersity [31]. Additionally, all characteristic peaks of BC and CuFe_2_O_4_ can be observed in the FT–IR spectra of fresh and used CuFe_2_O_4_@BC materials, demonstrating the successful synthesis of the designed materials and the basic structure of the material after the reaction was almost unchanged. After 30 min of reaction, the leaching rates of active components Fe and Cu are 0.087 mg/L and 0.214 mg/L, respectively (Table S1), which can be ignored, indicating that there was a strong interaction between CuFe_2_O_4_ and BC, and the CuFe_2_O_4_@BC material could still reach an 87.89% degradation rate of CIP after four cycles, revealing that it had strong stability. 

**Figure 3:**
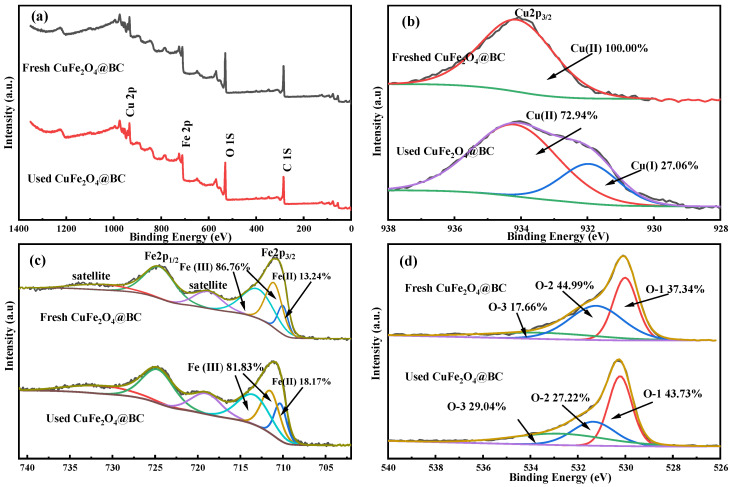
XPS spectroscopy wide scan (**a**) Cu 2p, (**b**) Fe 2p, (**c**) and O 1s, (**d**) of the fresh and used CuFe_2_O_4_@BC catalysts.

To explore the role of material surface components and several transition metals in the PMS activation process, the surface characteristics of CuFe_2_O_4_@BC material before and after the reaction were further studied by XPS. As shown in Figure 3, all binding energies were corrected with reference to C1s at 284.8 eV. The XPS spectroscopy wide scan of fresh and used CuFe_2_O_4_@BC material (Figure 3a) confirmed the coexistence of Cu, Fe, O, and C elements, which was consistent with the EDS spectrum results. Furthermore, Figure 3b demonstrated that the peak at 934.1 eV of fresh CuFe_2_O_4_@BC was assigned to the characteristic peak of Cu2p_3/2_, suggesting that Cu(II) was the main valence state of Cu species before catalysis. For the catalyst after reaction, a peak of 932.1 eV in the XPS Cu2p_3/2_ spectrum can be attributed to Cu(I) and its relative content was 27.06%, indicating that Cu(II) was partly reduced to Cu(I) after degradation. In Figure 3c, the Fe2p spectrum of fresh CuFe_2_O_4_@BC catalyst shows two peaks at 711.0 eV and 724.6 eV, assigning to Fe2p_3/2_ and Fe2p_1/2_, respectively, which represents that the Fe species on the CuFe_2_O_4_@BC surface was Fe(III) [34]. Notably, after participating in the degradation, the relative content of Fe(II) on the surface of the CuFe_2_O_4_@BC catalyst increased from 13.24% to 18.17%, whereas the relative content of Fe (III) decreased from 86.76% to 81.83%, indicating that Fe(III) was partially reduced to Fe(II) in the degradation process. Additionally, Figure 3d displayed the O 1s spectrum of fresh CuFe_2_O_4_@BC and used CuFe_2_O_4_@BC. Peaks at binding energies of 530.0, 531.3, and 532.9 eV were assigned to lattice oxygen (O-1), OH component (O-2), and the O of adsorbed water or carbonate (O-3), respectively [35]. After degradation, the significant decrease in O-2 relative content indicated that adsorbed O_2_ might participate in the catalytic degradation process, and the significant increase in O-1 and O-3 after degradation was assigned to the mineralization of pollutants. These results demonstrated the Fe(III)/Fe(II) and Cu(II)/Cu(I) circulation and the strong hydroxylation both occurred on the surface of the CuFe_2_O_4_@BC catalyst during the degradation process [36]. 

### 2.2. Catalytic Activity

**Figure 4:**
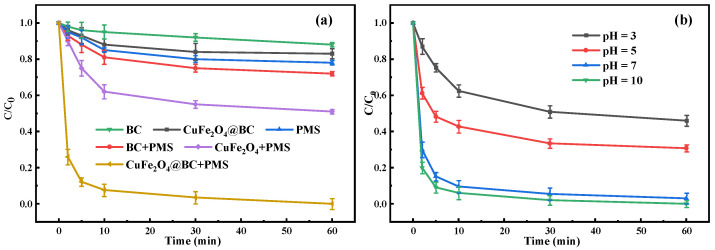
(**a**) CIP degradation efficiency activated by various catalysts. (**b**) Initial pH in the CuFe_2_O_4_@BC/PMS system. Reaction conditions: [CIP] = 10 mg/L, [PMS] = 2.5 mM, [catalysts] = 0.1 g/L (for **a**,**b**), initial pH = 7 (for **a**), initial pH = 3~10 (for **b**), temperature = 25 °C.

To systematically assess the catalytic activity of CuFe_2_O_4_@BC, a series of comparison tests with different catalysts were carried out to remove CIP. As shown in Figure 4a, the adsorption of CIP on the CuFe_2_O_4_@BC and BC surface was not obvious, and the CIP degradation rate was less than 20% during PMS oxidation alone. In addition, compared with the CuFe_2_O_4_/PMS and BC/PMS systems, the CuFe_2_O_4_@BC/PMS system had higher degradation activity for CIP, reaching a 98.72% degradation rate within 30 min. The CuFe_2_O_4_@BC exhibited higher catalytic activity for PMS than the traditional heterogeneous catalyst (Table S2). Previously reported that the activation of PMS was initiated by the electron transfer between PMS and the catalyst to produce reactive oxygen species (ROS) [37]. As a matrix rich in defect structure [25], BC could introduce a large number of oxygen vacancies (OVs) after compounding with CuFe_2_O_4_, which could be used as defect sites to promote the adsorption and bonding with PMS, indicating that the participation of Fe(III)/Fe(II) and Cu(II)/Cu(I) redox pairs [37] and the rich oxygen-containing functional groups on BC had synergistic effects on the activation of PMS to generate free radicals [26]. 

Furthermore, the leached metal ions in the two reaction systems were collected to evaluate the stability of the catalyst structure. As displayed in Table S1, in the CuFe_2_O_4_/PMS system (pH = 7), the dissolution concentrations of iron ions and copper ions were 0.455 and 1.279 mg/L at 30 min, respectively. However, the leaching of iron ions and copper ions reached the maximum concentration of 0.099 and 0.214 mg/L after 30 min in the CuFe_2_O_4_@BC/PMS system (pH = 7), which was far less than the leaching of metal ions in the CuFe_2_O_4_/PMS system. In contrast, the CuFe_2_O_4_@BC/PMS system had good stability for metal ion leaching, which might be attributed to the strong binding of CuFe_2_O_4_ to the BC matrix. According to SEM images in Figure 1a–c, CuFe_2_O_4_ particles were uniformly inserted in the BC basis. The unique structure decreased the leaching of metal ions, thereby, enhancing the synergistic effect and structural stability of CuFe_2_O_4_@BC.

### 2.3. Influences of Several Key Factors

#### 2.3.1. Influences of Initial Solution pH Value

The initial pH value of the reaction solution was a significant factor closely related to the catalytic performance of heterogeneous catalysts. Thus, we evaluated the effect of pH (3–10) on CIP removal. As presented in Figure 4b, the degradation efficiency increased from 54.3% to 100.0% as the solution pH value increased from 3.0 to 10.0. The efficient removal of CIP was achieved under neutral and weak alkali conditions, while the efficiency decreased dramatically in acidic conditions, which was consistent with previous studies [38,39]. Additionally, the zero-charge point of the material was 5.72 measured by the zetasizer, representing that the material surface carried a positive charge at pH < 5.72. Meanwhile, according to the pK_a1_ and pK_a2_ of PMS which are less than 0 and 9.4, respectively, PMS mainly exists in the form of HSO_5_^−^ under acidic conditions. However, previous studies have manifested that plenty of H^+^ would interact with the peroxide bond (-O-O-) of PMS to form hydrogen bonds, which inhibited its interaction with the positively charged CuFe_2_O_4_@BC surface by attaching the positive charge to HSO_5_^−^. Additionally, free radicals •OH, and SO_4_^•−^ can be eliminated by H^+^ (Equations (1) and (2)), which was also the course for the decrease in the CIP removal rate under acidic conditions [40].

•OH + H^+^ + e^−^ → H_2_O (1)

SO_4_^•−^ + H^+^ + e^−^ → HSO_4_^•−^ (2)

On the other hand, the remarkable CIP degradation efficiency under neutral and alkaline conditions was achieved in the CuFe_2_O_4_@BC/PMS system. Previous studies verified that the surface hydroxyl group was an important active species on the surface of heterogeneous catalysts, which could promote the chemical bonding between materials and PMS, thus, accelerating electron transfer [35]. Moreover, the increase in pH was conducive to the formation of surface hydroxyl groups in heterogeneous catalysts [41]. According to the strong coordination between phosphate and surface transition metal, which affects the formation of surface hydroxyl of the material [42], the experiment of adding 10 mM phosphate in the CuFe_2_O_4_@BC/PMS system was carried out to evaluate the influence of hydroxyl. As presented in Figure S3, with the addition of phosphate, the CIP degradation rate decreased from 100.0% to 22.05%, indicating that the surface hydroxyl plays a key role in the activation of PMS [41]. In addition, PMS could also directly generate superoxide radicals (O_2_^•−^) and singlet oxygen (^1^O_2_) [43]. For these reasons, the CuFe_2_O_4_@BC/PMS system realized the prominent degradation efficiency of CIP in both neutral and alkaline conditions. 

#### 2.3.2. Influences of Activator Loading and PMS Dosage

**Figure 5:**
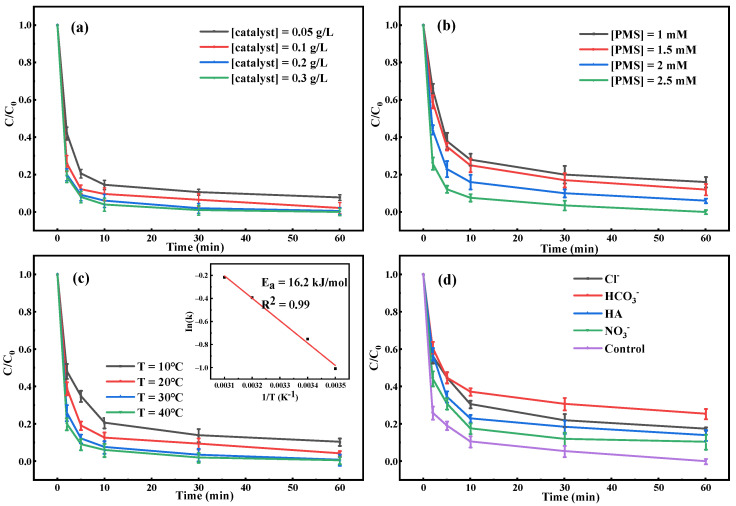
Influences of the (**a**) activator loading, (**b**) PMS dosage, (**c**) reaction temperature, and (**d**) various anions (10 mM) and HA (10 mg/L) on the CIP removal efficiency. Reaction conditions: [CIP] = 10 mg/L, [catalysts] = 0.1 g/L, [PMS] = 2.5 mM, initial pH = 7.0, T = 25 °C.

To preferably identify the activation efficiency of CuFe_2_O_4_@BC, the influences of activator loading and PMS dosage in the CuFe_2_O_4_@BC/PMS system were explored. As shown in Figure 5a, the CIP degradation rate was 92.22% within 60 min as the loading amount of the activator was 0.05 g/L. Additionally, when the loading increased from 0.1 g/L to 0.3 g/L, CIP could be degraded completely on CuFe_2_O_4_@BC within 60 min. A reasonable explanation might be that in the CuFe_2_O_4_@BC/PMS system when the amount of catalyst was too low, the active sites of PMS activation and CIP degradation tended to be insufficient [44]. Analogously, CIP degradation between 1 mM and 2.5 mM was positively dependent on PMS concentration in Figure 5b, which substantiated that the removal efficiency of CIP was limited on account of deficient PMS. 

#### 2.3.3. Influences of Reaction Temperature

In the CuFe_2_O_4_@BC/PMS system, the influence of reaction temperature was also investigated. As shown in Figure 5c, the degradation rate of CIP increases with an increasing reaction temperature. The initial reaction rate constants at 10, 20, 30, and 40 °C were calculated to be 0.3645 min^−1^, 0.4714 min^−1^, 0.6744 min^−1^, and 0.8052 min^−1^, respectively. Which was consistent with the previous reports that PMS could accelerate the generation of ROS under thermal activation [45]. Meanwhile, according to the Arrhenius equation (Equation (3)) based on the first-order kinetics at four different temperatures, the activation energy for the catalytic degradation of CIP by CuFe_2_O_4_@BC was calculated to be 16.2 kJ/mol (as illustrated in the inset of Figure 5c), which was much less than that of other studies (Table S3).

(3) $$\ln k=\ln A-\frac{Ea}{R}\left(\frac{1}{T}\right)$$

where *k* is the rate constant, *R* is the molar gas constant, *T* is the thermodynamic temperature, *Ea* is the apparent activation energy, and *A* is the pre-exponential factor.

#### 2.3.4. Influences of Chloride, Bicarbonate, Nitrate Ions, and Humic Acid (HA)

A variety of inorganic anions and natural organics such as humic acid are ubiquitous in actual wastewater, which can quickly eliminate ROS [46], thus, inhibiting the oxidation process. Accordingly, the effects of Cl^−^, HCO_3_^−^, NO_3_^−^, and HA on CIP removal efficiency were analyzed, as presented in Figure 5d. The degradation rate of CIP decreased to 81.52% with the presence of 5 mM Cl^−^ and the cause might be that Cl^−^ reacted with free radicals (•OH and SO_4_^•−^) to form chlorine-containing substances with high oxidation potential such as Cl^•^, Cl_2_^•−^, and ClOH^•−^ (k_Cl_^−^ _+ SO4_^•−^ = 3.1 × 10^8^ M^−1^ s^−1^, k_Cl_^−^ + _•OH_ = 4.3 × 10^9^ M^−1^ s^−1^) [25,47]. When the same amount of NO_3_^−^ was added, the inhibitory effect on CIP removal was less obvious, which was attributed to the fact that the NO_3_^−^ could scavenge SO_4_^•−^ with an extremely small reaction rate (5.0 × 10^4^ M^−1^ s^−1^) [48]. Whereas, when 5 mM HCO_3_^−^ was added, the removal efficiency of the reaction system decreased to 73.87% after 60 min. According to previous reports, HCO_3_^−^ could react with •OH and SO_4_^•−^ at the rate of 8.5 × 10^6^ M^−1^ s^−1^ and 3.6 × 10^6^ M^−1^ s^−1^ [49]. The oxidation activities of the generated CO_3_^•−^ was lower than that of •OH and SO_4_^•−^, which demonstrated that HCO_3_^−^ had an obvious inhibitory effect [25]. In addition, the addition of 5 mM humic acid (HA), which represented natural organic matter in the aquatic system, decreased the degradation efficiency to 86.89% with a marginal inhibition of the catalytic activity. This might be due to the competitive effect of CIP and HA on ROS [49].

### 2.4. Magnetism and Reusability of the Catalyst

**Figure 6:**
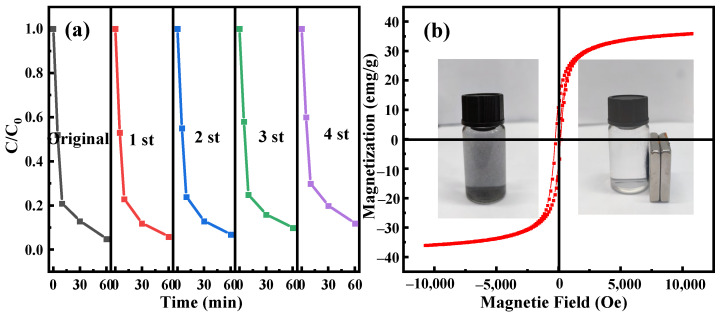
(**a**) Cycle experiments. (**b**) Optical photographs of magnetic separation process and magnetic properties of the CuFe_2_O_4_@BC catalyst. Reaction conditions: [CIP] = 10 mg/L, [catalysts] = 0.1 g/L, [PMS] = 2.5 mM, initial pH = 7.0, T = 25 °C.

As presented in Figure 6a, CIP degradation efficiency could still achieve 88.01% after five circulations. The decrease in the CIP removal efficiency might be assigned to the loss of active ingredients. Meanwhile, the copper–iron ion leaching experiment showed that 0.087 mg/L iron ion and 0.214 mg/L copper ion were detected at 30 min, which was insignificant for the overall degradation process. The interaction between graphitized biochar and CuFe_2_O_4_ particles enhances the stability of the overall structure. Furthermore, the measured saturation magnetization value of the material 35.78 emu/g was displayed in Figure 6b, suggesting that CuFe_2_O_4_@BC had noteworthy magnetism and the CuFe_2_O_4_@BC material was easily recovered from the reaction solution according to the illustration in Figure 6b. Simply implement an external magnetic field without a cumbersome separation process.

### 2.5. Investigation of Reactive Species in CuFe_2_O_4_@BC/PMS System 

**Figure 7:**
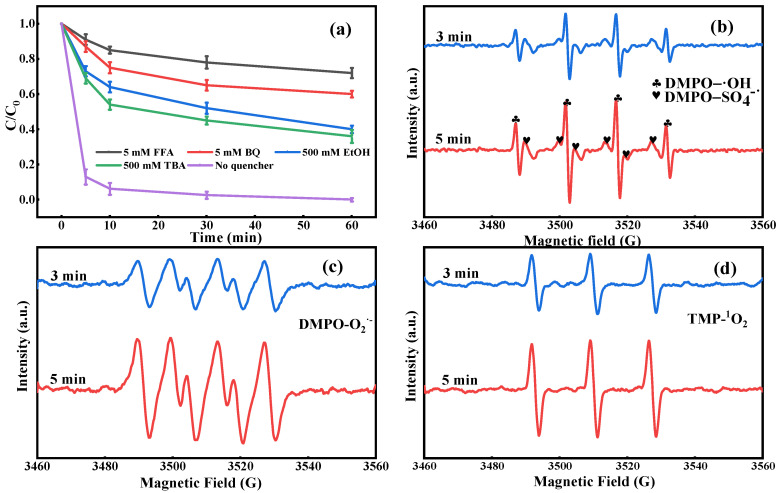
(**a**) Effect of different scavengers on the degradation of CIP and EPR spectra of CuFe_2_O_4_@BC/PMS system (**b**) DMPO for SO_4_^•−^ and •OH, TEMP for (**c**) O_2_^•−^ and (**d**) ^1^O_2_. Reaction conditions: [CIP] = 10 mg/L, [catalysts] = 0.1 g/L, [PMS] = 2.5 mM, initial pH = 7.0, T = 25 °C.

To testify the types of ROS generated in the CuFe_2_O_4_@BC/PMS system and their contribution to the catalytic reaction, free radical quenching experiments were performed. Accordingly, the reaction rate constants of EtOH, TBA, BQ, and FFA with different ROS are summarized in Table S4. With the high reaction rate constants, EtOH was adopted as a quencher of both SO_4_^•−^ and •OH. In contrast, TBA mainly quenched •OH and shows a weak ability to react with SO_4_^•−^. Thus, TBA acted as an •OH scavenger. Additionally, BQ can react with O_2_^•−^ at a high reaction rate, which was selected as an effective scavenger of O_2_^•−^ to ascertain the role of O_2_^•−^ in the CuFe_2_O_4_@BC/PMS system. Owing to both TBA and FFA exhibiting the same quenching ability •OH and FFA showed an effective scavenging effect on ^1^O_2_, thus, FFA was chosen as a scavenger to explore the effect of ^1^O_2_ in the CuFe_2_O_4_@BC/PMS system. As displayed in Figure 7a, after adding 500 mM EtOH and 500 mM TBA, the CIP degradation rate decreased slightly from 100.0% to 60.2% and 64.1%, respectively. In contrast, the CIP degradation rate decreased significantly from 100.0% to 36.3% with the addition of 5 mM BQ. It is noteworthy that BQ can not only quench O_2_^•−^ efficiently but also react with •OH and SO_4_^•−^, compared with the decrease rate of the CIP degradation rate after adding EtOH. The addition of 5 m M BQ observably inhibited the degradation of CIP, ∼63.8% inhibition of CIP degradation efficiency could be obtained. Moreover, the addition of 5 mM FFA prominently reduced the performance of the composite and the CIP degradation rate decreased from 100.0% to 27.4%, which manifested a more remarkable ability to capture free radicals compared with the addition of 500 mM TBA. Considering the high inhibition efficiency of O_2_^•−^ and ^1^O_2_, different concentrations of BQ and FFA were added to the reaction solution to verify the exact effect of O_2_^•−^ and ^1^O_2_ (Figure S4). The results manifested that the CIP degradation efficiency was remarkably inhibited with increasing BQ concentration from 1 to 5 mM, and the degradation efficiency of CIP also decreased with the addition of higher concentrations of FFA, indicating the important role of O_2_^•−^ and ^1^O_2_ in the CuFe_2_O_4_@BC/PMS system.

To further identify the production of SO_4_^•−^, •OH, O_2_^•−^, and ^1^O_2_, EPR was employed to detect the radical generation by coupling spin trapping agents DMPO and TEMP. Expectedly, the characteristic signals of DMPO-•OH adducts (a typical signals of 1:2:2:1 quartet peaks with hyperfine coupling constants of α_N_ = 14.86 G, α_H_ = 14.86 G) and DMPO-SO_4_^•−^ adducts (α_H_ = 1.44 G, α_H_ = 0.76 G, α_N_ = 15.01 G, and α_H_ = 14.82 G) were detected in Figure 7b; therefore, the SO_4_^•−^ and •OH were considered to exist in the CuFe_2_O_4_@BC/PMS system. Analogously, the typical spectral line of DMPO-O_2_^•−^ EPR signals was also clearly identified in Figure 7c, indicating that the O_2_^•−^ was generated in the degradation process. Additionally, as presented in Figure 7d, the typical equal triplet signal attributed to the characteristic TEMP-^1^O_2_ adducts certify the existence of ^1^O_2_ in the CuFe_2_O_4_@BC/PMS system. Based on the above-mentioned analysis, SO_4_^•−^, •OH, O_2_^•−^, and ^1^O_2_ were all involved in the degradation of CIP, but O_2_^•−^ and ^1^O_2_ were recognized as the dominant ROS in CuFe_2_O_4_@BC/PMS system.

### 2.6. Proposed Activating Mechanism of CuFe_2_O_4_@BC

**Figure 8:**
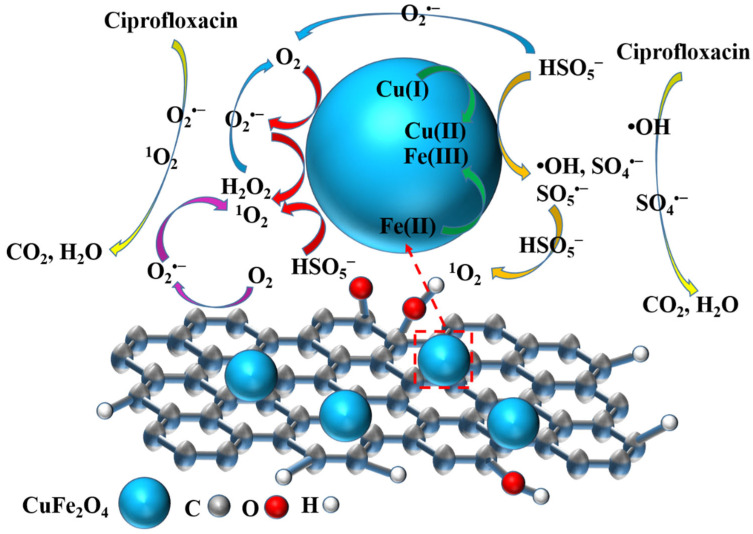
Proposed degradation mechanism of CuFe_2_O_4_@BC/PMS system.

Based on the aforementioned analyses, the proposed catalytic degradation mechanism in the CuFe_2_O_4_@BC/PMS system was presented in Figure 8. During the activating process, the peroxy bond (-O-O-) of PMS can be broken through the Fe(III)/Fe(II) and Cu(II)/Cu(I) circulation to generate SO_4_^•−^ and •OH (Equations (4) and (5)) and the Fe(III)/Cu(II) could react with PMS to produce SO_5_^•−^ with activity lower than SO_4_^•−^ (Equation (6)). Furthermore, according to the previous quenching tests [50] combined with EPR analysis, ^1^O_2_ and O_2_^•−^ play important roles in the system. Based on the previous literature reports and the experiment results obtained in this paper, it could be inferred that there were two generation processes of the O_2_^•−^ in the CuFe_2_O_4_@BC/PMS system: (i) the defect structures of biochars could facilitate the electron transfer of O_2_ and then produce O_2_^•−^ (Equation (7)); (ii) with the assistance of the abundant OVs in the CuFe_2_O_4_ crystal, electrons are transferred to O_2_ through CuFe_2_O_4_ and then reduced to O_2_^•−^ (Equation (8)). In addition, O_2_^•−^ could not only promote the generation of ^1^O_2_ (Equations (9) and (10)) [51] but also facilitate the decomposition of PMS to generate SO_4_^•−^ and •OH (Equations (11) and (12)). Then the O_2_ reacted during the degradation process and could be supplemented by Equations (11)–(13). Finally, the produced ROS attacked CIP and degraded into intermediates, which might even be mineralized into CO_2_ and H_2_O (Equation (14)). Therefore, BC could improve the dispersion of CuFe_2_O_4_ which enhances the structural stability of the catalyst and also facilitates electron conversion due to the excellent electrical conductivity, showing the synergistic effects between CuFe_2_O_4_ and BC that could effectively catalyze PMS to degrade CIP.

Cu(I)/Fe(II) + HSO_5_^−^ → SO_4_^2−^ + •OH + Cu(II)/Fe(III) (4)

Cu(I)/Fe(II) + HSO_5_^−^ → SO_4_^•−^ + OH^−^ + Cu(II)/Fe(III) (5)

Cu(II)/Fe(III) + HSO_5_^−^ → SO_5_^•−^ + H^+^ + Cu(I)/Fe(II) (6)

BC + O_2_ → O_2_^•−^ + BC^+^ (7)

Cu(I)/Fe(II) + O_2_ → O_2_^•−^ + Cu(II)/Fe(III) (8)

2O_2_^•−^ + 2H^+^ → ^1^O_2_ + H_2_O_2_ (9)

•OH + O_2_^•−^ → ^1^O_2_ + OH^−^ (10)

O_2_^•−^ + H_2_O_2_ → O_2_ + 2•OH (11)

O_2_^•−^ + HSO_5_^−^ → O_2_ + SO_4_^•−^ + OH^−^ (12)

2SO_5_^•−^ → 2SO_4_^•−^ + O_2_ (13)

ROS + CIP → Intermediates → CO_2_ + H_2_O (14)

## 3. Material and Method

### 3.1. Chemical Reagents

Oxone (PMS, KHSO_5_·0.5KHSO_4_·0.5K_2_SO_4_) was obtained from Sigma-Aldrich. Iron nitrate nonahydrate (Fe(NO_3_)_3_·9H_2_O), citric acid, copper nitrate trihydrate (Cu(NO_3_)_2_·3H_2_O), ciprofloxacin (CIP), tert-butyl alcohol (TBA), potassium hydroxide (KOH), and hydrochloric acid (HCl) were supplied by Shanghai Macklin Biochemical Co. Ltd. (Shanghai, China). Sodium nitrate (NaNO_3_) was purchased from Tianjin Kermel Chemical Reagent Co. Ltd. (Tianjin, China). Other reagents, including benzoquinone (BQ), humic acid (HA), sodium bicarbonate (NaHCO_3_), ethanol (EtOH), methanol (HPLC grade), superoxide dismutase (SOD), and sodium chloride (NaCl) were purchased from Aladdin Bio-Chem Technology Co. Ltd. (Shanghai, China). All reagents were at least analytical grade. The deionized (DI) water was produced in the laboratory with resistivity >10 MΩ cm. Waste mulberry branches come from the College of Sericulture Textile and Biomass Science, Southwest University, China. 

### 3.2. Preparation of CuFe_2_O_4_@BC Catalyst

After being washed with deionized water several times, the waste mulberry branches were dried at 90 °C, ground into particles, and then pyrolyzed under oxygen-limited conditions for 2 h at 700 °C, at a rate of 10 °C min^−1^. After cooling, the obtained biochar was ground and sieved for later use.

CuFe_2_O_4_@BC was prepared by the modified sol-gel method as reported in the literature [52]. In brief, 2.020 g Fe(NO_3_)_3_·9H_2_O, 0.605 g Cu(NO_3_)_2_·3H_2_O, and 0.6 g prepared BC were firstly mixed in 50 mL DI water (the mass ratio of CuFe_2_O_4_/BC was optimized to be 1:1). Then the mixture was stirred in an oil bath for 3 h at 60 °C, added 3.75 g citric acid, and continue to stir for another 3 h to form homogeneous dispersion. After that, subjected the mixture to a vacuum oven to remove water at 100 °C. The resulting black sticky gel was injected into a porcelain crucible and heated in a tube furnace at 700 °C for 2 h at a heating rate of 10 °C/min in an N_2_ atmosphere (60 mL/min). Then, the obtained material was washed to neutral with 1 M KOH and 2 M HCl to remove the residual impurities. Finally, the catalyst was dried at 90 °C. For comparison, naked CuFe_2_O_4_ was also synthesized under the same conditions except for no addition of BC.

### 3.3. Catalyst Characterization

The structural morphologies and particle sizes of the as-prepared catalyst were obtained by the ultra-high resolution transmission scanning electron microscope (SEM; SU8020, Hitachi Limited, Tokyo, Japan) and the transmission electron microscope (TEM; FEI Talos F200S, FEI Company, Hillsboro, OR, USA). Types and contents of elements in material micro-area composition were analyzed by an X-ray energy dispersive spectrometer (EDS; HORIBA EX-350, Hitachi Limited, Tokyo, Japan ). The crystal structures and phases of as-prepared catalysts were characterized by an X-ray diffractometer (XRD, XD-6, Beijing Pgeneral, Beijing, China) using Cu-Kα radiation (36 kV, 20 mA). The chemical bonds and functional groups of samples were identified by Fourier transform infrared spectrometer (FTIR, Nicolet IS10, Waltham, MA, USA). X-ray photoelectron spectroscopy (XPS; Escalab 250Xi, Thermo Fisher Scientific, Inc., Waltham, MA, USA) was used to detect the surface chemical states and compositions of the elements of catalyst. The magnetic property of the CuFe_2_O_4_@BC catalyst was characterized by a vibrating sample magnetometer (SQUID-VSM, MPMS-3, Quantum Design, San Diego, CA, USA). Zetasizer Nano-ZS90 (Malvern, UK) was used to identify the point of zero charge (pH_pzc_) of CuFe_2_O_4_@BC.

### 3.4. Catalytic Activity Experiment

Batch experiments were performed in a 300 mL conical flask under shaking in a table concentrator at 25 ± 1.0 °C. The initial concentration of 2.5 mM PMS was fully dispersed into a 100 mL 10 mg/L CIP aqueous solution. Then, after adding 10 mg of catalyst, the degradation reaction was initiated and the samples were withdrawn at a specified time interval, filtered by a 0.22 μm filter membrane, quenched by 0.2 mL anhydrous ethanol for further analysis. After each run, the CuFe_2_O_4_@BC was recovered by the external magnetic field, washed with DI water, dried at 60 °C, and reused for the other four cycles. All experiments were carried out at least three times to guarantee the accuracy. 

### 3.5. Analytical Methods

The leachable iron and copper ions after degradation were obtained from the inductively coupled plasma optical emission spectrometer (ICP-OES, Agilent Technologies Inc., Santa Clara, CA, USA). Furthermore, CIP was analyzed by high-performance liquid chromatography (HPLC; Shimadzu LC-20AT, Kyoto, Japan), which is composed of a C18 column (4.6 mm) and an SPD-20A detector (267 nm). The column temperature was maintained at 30 °C, and the mobile phases are 70% water (A) and 30% methanol (B), the flow rate was set to 1 mL/min. In addition, the electron paramagnetic resonance (EPR, Bruker A300-10/12, Ettlingen, Germany) was used to detect ROS captured by DMPO and TMP. 

## 4. Conclusions

In summary, the CuFe_2_O_4_@BC composite was successfully synthesized by a modified sol-gel calcination method and characterized by a series of characterization measurements, namely SEM, XRD, FTIR, XPS, and VSM. Noticeably, the CuFe_2_O_4_@BC catalyst presented remarkable catalytic activity and stability, contributing to the significant degradation efficiency of CIP. Furthermore, the CIP degradation efficiency was enhanced with the increase in the activator loading (0.05–0.3 g/L), PMS dosage (1–2.5 mM), and reaction temperature (10–40 °C), while the effect of the initial solution pH on the removal efficiency was the opposite. Meanwhile, the addition of Cl^−^, NO_3_^−^, HCO_3_^−^ anions, and HA restrained the removal efficiency to varying degrees and CuFe_2_O_4_@BC also showed superior reusability due to its stable structure and remarkable magnetic properties. Moreover, quenching experiments combined with EPR examination implied that SO_4_^•−^, •OH, O_2_^•−^, and ^1^O_2_ existed in the degradation process, and ^1^O_2_ and O_2_^•−^ were identified as the dominant ROS. The synergistic effect between CuFe_2_O_4_ and BC enhanced the structural stability and electrical conductivity of the material, which promoted the bonding between the catalyst and PMS, as well as accelerated the circulation of Fe(III)/Fe(II) and Cu(II)/Cu(I), resulting in the improved catalytic efficiency of CuFe_2_O_4_@BC. This paper emphasized the potential of CuFe_2_O_4_@BC composite for PMS activation towards the degradation of organic pollutants, which might be used as a promising wastewater remediation strategy. 

## Data Availability

Not applicable.

## Supplementary Materials

The following supporting information can be downloaded at: https://www.mdpi.com/article/10.3390/ijms24065702/s1. References [20,37,39,42,45,50,53,54,55,56,57,58,59] are cited in the supplementary materials.
