# Supplementary material for: Catalytic Degradation of Ciprofloxacin in Aqueous Solution by Peroxymonosulfate Activated with a Magnetic CuFe2O4@Biochar Composite
Source: Int J Mol Sci. 2023 Mar 16;24(6):5702. doi: 10.3390/ijms24065702 (PMC10051636; doi:10.3390/ijms24065702)
Supplement: Supplementary file 1 [file ijms-24-05702-s001.zip › ijms-2198973-supplementary.pdf]

Supporting Information

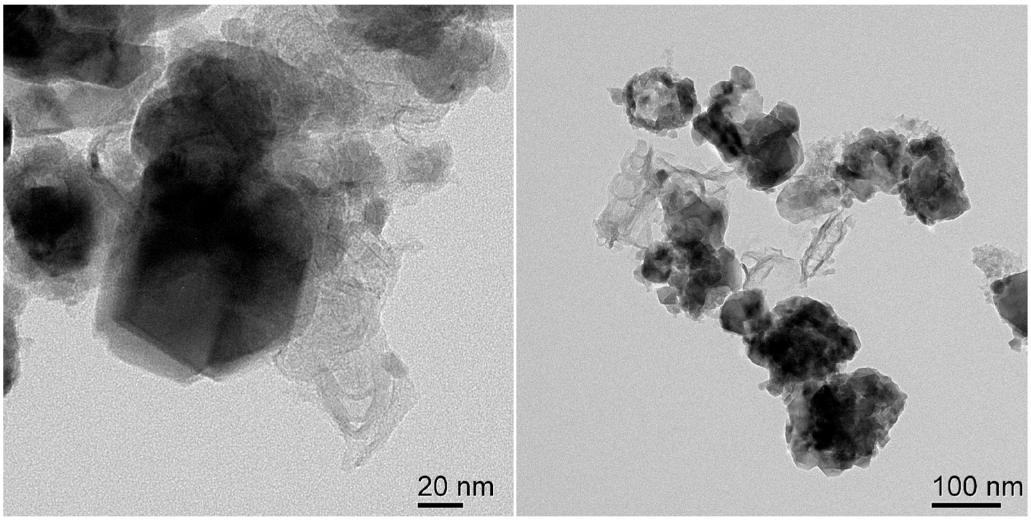

Figure S1. Representative TEM image of the $CuFe_2O_4$@BC particles.

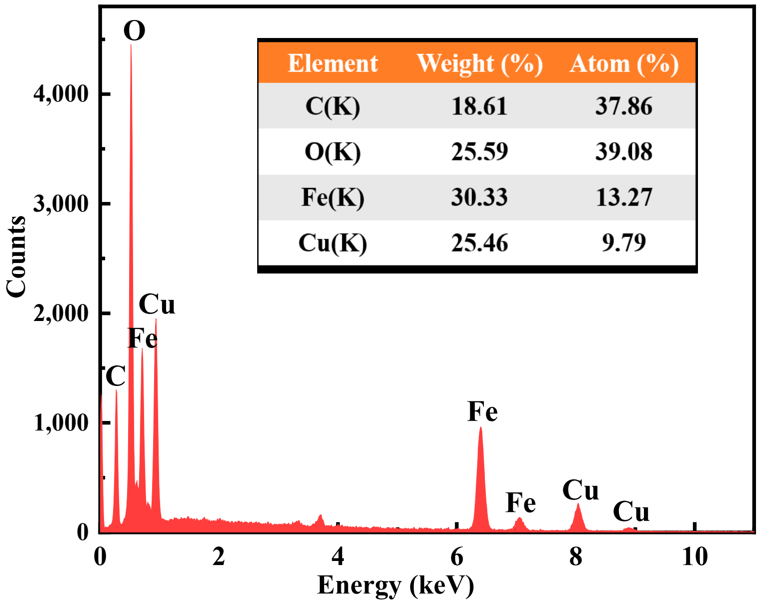

Figure S2. EDS spectrum of the $CuFe_2O_4$@BC.

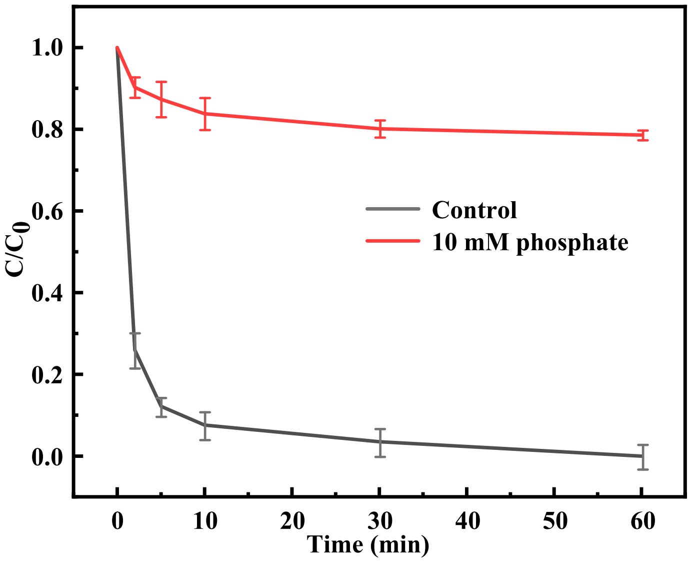

Figure S3. CIP degradation with adding 10 mM phosphate ions in $CuFe_2O_4$@BC/PMS system. Reaction conditions: [CIP] = 10 mg/L, [PMS] = 2.5 mM, [catalysts] = 0.1 g/L, initial pH = 7.0, temperature = 25 °C.

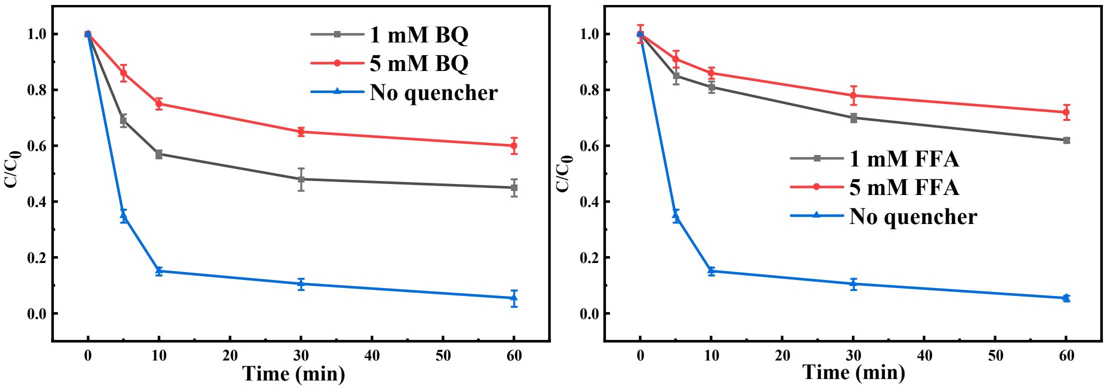

Figure S4. CIP degradation with adding different concentrations of BQ and FFA in $CuFe_2O_4$@BC/PMS system. Reaction conditions: [CIP] = 10 mg/L, [PMS] = 2.5 mM, [catalysts] = 0.1 g/L, initial pH = 7.0, temperature = 25 °C.

Table S1. Concentrations of leached metal ions from different reaction conditions.

Conditions	Copper Ions (mg/L)	Iron Ions (mg/L)
$CuFe_2O_4$@BC (pH=3)	0.363	0.105
$CuFe_2O_4$@BC (pH=7)	0.214	0.087
$CuFe_2O_4$ (pH=3)	1.290	0.525
$CuFe_2O_4$ (pH=7)	1.279	0.455

Table S2. Comparison of the reaction parameters with previously reported catalysts for PMS activation.

Pollution	Catalysts Loading	PMS Dosage	Removal Efficiency	Reference
PCB28 (0.5 mg L^{-1})	$CuFe_2O_4$ (0.1 g L^{-1})	0.2 mM	89% (8 h)	[51]
Phenol (35 mg L^{-1})	Cu-MnO_2 (0.3 g L^{-1})	0.4 g L^{-1}	100% (40 min)	[53]
methylene blue (20 mg L^{-1})	$CuFe_2O_4$-AC (0.2 g L^{-1})	2.0 g L^{-1}	100% (2 h)	[54]
CIP (10 mg L^{-1})	ZCFO (0.1 g L^{-1})	2.5 mM	96.6% (15 min)	[39]
BPS (10 μM)	$CuCo_2S_4$ (0.01 g L^{-1})	100 μM	100% (30 min)	[55]
BPA (10 mg L^{-1})	$Mn_{1.8}Fe_{1.2}O_4$ (0.1 g L^{-1})	0.2 g L^{-1}	95% (30 min)	[43]
4-CP (100 μM)	Co-Black TNT	1 mM	100% (30 min)	[38]
CIP (10 mg L^{-1})	$CuFe_2O_4$@BC (0.1 g L^{-1})	2.5 mM	97.8% (30 min)	Our work

Table S3. Comparison of activation energy of CIP degradation with previously reported catalysts.

Entry	System	Catalysts loading	Oxidant dosage	Ea	Reference
1	nano-Co_3O_4/PMS	0.1 g L^{-1}	1 mM	61.9 kJ/mol	[46]
2	Co@N-C/PMS	0.1 g L^{-1}	0.25 mM	35.4 kJ/mol	[56]
3	$MnFe_2O_4$/PMS	0.05 g L^{-1}	0.5 g L^{-1}	31.7 kJ/mol	[57]
4	$CuFe_2O_4$@BC/PMS	0.1 g L^{-1}	2.5 mM	16.2 kJ/mol	Our work

Table S4. Summary on the second-order reaction rates of EtOH, TBA, FFA, BQ and the first-order reaction rates of BQ with different ROS.

Scavengers	Second-order reaction rata constants($M^{-1}s^{-1}$)[20,58]			
	•OH	$SO_4^{\bullet-}$	$O_2^{\bullet-}$	$^1O_2$
EtOH	1.9×10^9	$(1.6\text{-}7.7) \times 10^7$	$< 10^3$	$3.8 \text{ x } 10^3$
TBA	$(3.8\text{-}7.6)\ 10^8$	$(4.0\text{-}8.1) \times 10^5$	$< 10^3$	$< 10^4$
FFA	1.5×10^{10}	No reaction	No reaction	1.2×10^8
BQ	$1.2\ 10^9$	1.0×10^8	$(0.9\text{-}1.9) \times 10^9$	6.6×10^7
**Scavengers**	**First-order reaction rata constants($M^{-1}s^{-1}$)[59]**			
	•OH	$SO_4^{\bullet-}$	$O_2^{\bullet-}$	$^1O_2$
BQ	4.8×10^6	4.0×10^5	$(3.6\text{-}4.0) \times 10^6$	1.52×10^5

References:

51. Qin, W.; Fang, G.; Wang, Y.; Zhou, D. Mechanistic understanding of polychlorinated biphenyls degradation by peroxymonosulfate activated with CuFe2O4 nanoparticles: Key role of superoxide radicals. *Chemical Engineering Journal* **2018**, *348*, 526-534, doi:10.1016/j.cej.2018.04.215.
53. Huang, Y.; Tian, X.; Nie, Y.; Yang, C.; Wang, Y. Enhanced peroxymonosulfate activation for phenol degradation over MnO2 at pH 3.5-9.0 via Cu(II) substitution. *Journal of hazardous materials* **2018**, *360*, 303-310, doi:10.1016/j.jhazmat.2018.08.028.
54. Oh, W.D.; Lua, S.K.; Dong, Z.; Lim, T.T. Performance of magnetic activated carbon composite as peroxymonosulfate activator and regenerable adsorbent via sulfate radical-mediated oxidation processes. *Journal of hazardous materials* **2015**, *284*, 1-9, doi:10.1016/j.jhazmat.2014.10.042.
39. Yu, R.; Zhao, J.; Zhao, Z.; Cui, F. Copper substituted zinc ferrite with abundant oxygen vacancies for enhanced ciprofloxacin degradation via peroxymonosulfate activation. *Journal of hazardous materials* **2020**, *390*, 121998, doi:10.1016/j.jhazmat.2019.121998.
55. Xu, H.; Wang, D.; Ma, J.; Zhang, T.; Lu, X.; Chen, Z. A superior active and stable spinel sulfide for catalytic peroxymonosulfate oxidation of bisphenol S. *Applied Catalysis B: Environmental* **2018**, *238*, 557-567, doi:10.1016/j.apcatb.2018.07.058.
43. Huang, G.-X.; Wang, C.-Y.; Yang, C.-W.; Guo, P.-C.; Yu, H.-Q. Degradation of Bisphenol A by Peroxymonosulfate Catalytically Activated with Mn1.8Fe1.2O4 Nanospheres: Synergism between Mn and Fe. *Environmental science & technology* **2017**, *51*, 12611-12618, doi:10.1021/acs.est.7b03007.
38. Lim, J.; Yang, Y.; Hoffmann, M.R. Activation of Peroxymonosulfate by Oxygen Vacancies-Enriched Cobalt-Doped Black TiO2 Nanotubes for the Removal of Organic Pollutants. *Environmental science & technology* **2019**, *53*, 6972-6980, doi:10.1021/acs.est.9b01449.
46. Deng, J.; Feng, S.; Zhang, K.; Li, J.; Wang, H.; Zhang, T.; Ma, X. Heterogeneous activation of peroxymonosulfate using ordered mesoporous Co 3 O 4 for the degradation of chloramphenicol at neutral pH. *Chemical Engineering Journal* **2017**, *308*, 505-515, doi:10.1016/j.cej.2016.09.075.
56. Li, H.; Tian, J.; Zhu, Z.; Cui, F.; Zhu, Y.-A.; Duan, X.; Wang, S. Magnetic nitrogen-doped nanocarbons for enhanced metal-free catalytic oxidation: Integrated experimental and theoretical investigations for mechanism and application. *Chemical Engineering Journal* **2018**, *354*, 507-516, doi:10.1016/j.cej.2018.08.043.
57. Zhu, L.; Shi, Z.; Deng, L.; Duan, Y. Efficient degradation of sulfadiazine using magnetically recoverable MnFe2O4/δ-MnO2 hybrid as a heterogeneous catalyst of peroxymonosulfate. *Colloids and Surfaces A: Physicochemical and Engineering Aspects* **2021**, *609*, 125637, doi:10.1016/j.colsurfa.2020.125637.
20. Zhang, T.; Zhu, H.; Croué, J.-P. Production of Sulfate Radical from Peroxymonosulfate Induced by a Magnetically Separable CuFe2O4 Spinel in Water: Efficiency, Stability, and Mechanism. *Environmental science & technology* **2013**, *47*, 2784-2791, doi:10.1021/es304721g.
58. Duan, X.; Sun, H.; Wang, Y.; Kang, J.; Wang, S. N-Doping-Induced Nonradical Reaction on Single-Walled Carbon Nanotubes for Catalytic Phenol Oxidation. *ACS Catalysis* **2014**, *5*, 553-559, doi:10.1021/cs5017613.
59. Wang, Y.; Cao, D.; Zhao, X. Heterogeneous degradation of refractory pollutants by peroxymonosulfate activated by CoOx-doped ordered mesoporous carbon. *Chemical Engineering Journal* **2017**, *328*, 1112-1121, doi:10.1016/j.cej.2017.07.042.
